# The Pre-Exposure Prophylaxis-Stigma Paradox: Learning from Canada's First Wave of PrEP Users

**DOI:** 10.1089/apc.2017.0153

**Published:** 2018-01-01

**Authors:** Daniel Grace, Jody Jollimore, Paul MacPherson, Matthew J.P. Strang, Darrell H.S. Tan

**Affiliations:** ^1^ University of Toronto, Dalla Lana School of Public Health, Toronto, Ontario, Canada.; ^2^ Community-Based Research Centre for Gay Men's Health, Vancouver, British Columbia, Canada.; ^3^ Ottawa Hospital Research Institute, Ottawa, Ontario, Canada.; ^4^ York University, Department of Sociology, Toronto, Ontario, Canada.; ^5^ St. Michael's Hospital, Toronto, Ontario, Canada.

**Keywords:** pre-exposure prophylaxis, HIV prevention, gay men, sexual stigma, sexual risk

## Abstract

With the emergence of daily oral tenofovir disoproxil fumarate and emtricitabine-based pre-exposure prophylaxis (PrEP) use in Canada, questions have emerged concerning the impacts of this HIV prevention tool on gay men's social and sexual lives. We conducted small focus groups and individual qualitative interviews with 16 gay men in Toronto who were part of the ‘first wave’ of Canadian PrEP users. Participants were on PrEP for at least one year as part of a demonstration project (November 2014–June 2016). These participants accessed PrEP before regulatory approval by Health Canada in February 2016. The mean age of participants was 37.6 years (SD 11.02); 94% completed secondary education, and 69% were white. Sex-stigma emerged as a complex theme in men's accounts of PrEP use across three overlapping domains: (1) *PrEP-related stigma*, including discussions of concealment and stigma from friends, family, and sexual partners, (2) *PrEP as a perceived tool for combating HIV-related stigma*, where some men said that they no longer discussed HIV status with sexual partners, and (3) *PrEP as illuminating structural stigma*, where it was attributed to unmasking stigma related to sex and sexuality. For some participants, PrEP has allowed for *liberating* sex and a self-described *return to normalcy*—normal, exciting, pleasurable sex that was no longer reliant on condom use. Paradoxically, some men said that PrEP use both led them to *experience stigmatizing reactions* within their social and sexual networks, while also helping to *remove stigma, shame, and fear* related to HIV, sexuality, and sex with gay men living with HIV.

## Introduction

Canada continues to see unrelenting rates of HIV transmission, with a persistently disproportionate burden of new infections occurring among gay, bisexual, and other men who have sex with men (gbMSM). This diverse group makes up 54.3% of incident HIV infections, with the relative risk of contracting HIV being 131 times higher than other Canadian men, estimated at 469 per 100,000 individuals in 2014.^1,2^ To help address the HIV epidemic among gbMSM, there has been increasing interest in the use of HIV pre-exposure prophylaxis (PrEP) as part of a combination approach to reduce transmission.^3,4^ PrEP refers to the daily oral use of the antiretroviral medications tenofovir disoproxil fumarate and emtricitabine (TDF/FTC) by HIV uninfected persons, who are at ongoing risk, to block infection before it occurs. Clinical trials and observational studies have demonstrated the high efficacy of PrEP for preventing HIV among gbMSM when adherence is high, conferring a risk reduction of 100% (95% confidence interval, 86–100%) when 4–7 doses of daily PrEP are taken each week.^5–7^

PrEP has been prescribed off-label in Canada for several years by a small number of specialists, with usage increasing steadily since regulatory approval was granted by Health Canada in February 2016. Medication costs have been the primary barrier to more widespread usage, as PrEP has generally not been covered by most public drug plans nationwide, and a 30-tablet bottle of brand-name PrEP (Truvada^®^) can cost between $850 to $1000 CAD in different parts of Canada. However, the recent entry of generic TDF/FTC onto the Canadian market has decreased prices to roughly 25% of this amount as of late 2017, and policies regarding public reimbursement are under review. As of late September 2017, Ontario (Canada's most populous province, and the province in which this work was conducted) added TDF/FTC for PrEP onto the provincial drug formulary, making it accessible for free to individuals on social assistance or aged ≥65 years, or for a modest deductible (3–4% of household after-tax income).^8^

PrEP, along with knowledge of other biomedical advancements in the evolution of HIV prevention (e.g., reduced HIV transmission risk due to antiretroviral use among HIV-positive persons), has also raised questions concerning how gbMSM understand increasingly complex health information, and the extent to which knowledge of HIV transmission risk informs prevention repertoires and sexual decision-making.^9^ For example, Grace et al. have used longitudinal qualitative interviews to examine how having an undetectable viral load was experienced as a significant milestone and emergent prevention identity for many gay men recently diagnosed with HIV.^10^ Participants described that becoming “undetectable” served as a significant goal to achieve, as a category enabling both sexual possibilities and rejections, and as a signifier of a return to normalcy postdiagnosis.

Beyond specific questions of PrEP efficacy and implementation, Auerbach and Hoppe argue that “PrEP raises a number of other important social-psychological questions that also must be attended to in order to translate biomedical and clinical findings into uptake of PrEP among enough people at risk of HIV infection to produce population-level effectiveness.”^11^ Limited qualitative and mixed methods research has been conducted to date with early prescribers^12^ and adopters^13,14^ (or *potential* candidates^15,16^) of this HIV prevention intervention.

The current study was conducted to inductively learn from the lived experiences of PrEP users who were part of the PREPARATORY-5 study in Toronto, Canada, and had been on PrEP for a year or longer. In-depth interviews allowed us to use social science research methods to ask about the everyday social and sexual realities of PrEP use, including PrEP-related barriers^17^ and psychosocial challenges faced by gay men who were some of the earliest adopters of this new biomedical HIV prevention tool in Canada. These interviews allowed us to explore the everyday actualities of navigating one's sexuality and sexual partnerships in a shifting HIV prevention context.

## Methods

### Recruitment

All participants in this qualitative study were recruited from the PREPARATORY-5 demonstration project in Toronto, which ran from November 2014 to June 2016.^18^ PREPARATORY-5 was an open-label, 12-month demonstration project at an academic hospital-based HIV clinic in downtown Toronto, whose main objectives were to obtain pilot data on PrEP acceptability and clinical outcomes among Toronto gbMSM (NCT02149888). To be eligible, gbMSM had to be 18 years or older, living in the greater Toronto area, HIV negative, and at high risk for HIV acquisition (defined as scoring ≥10 on the HIRI-MSM and engaging in least 1 act of condomless receptive anal sex within the past 6 months).^19^ The target sample size for the pilot project was 50.

Fifty-two gbMSM were ultimately enrolled, and were provided with 12 months of free TDF/FTC. After the study was completed, the trial participants who had consented to being contacted about future research were invited by clinic staff to participate in qualitative interviews. We successfully recruited 16 trial participants to take part in these interviews. Participants were given the option of taking part in small focus group interviews or individual interviews. In all but one instance, where the participant did not want to participate in a group interview format, individual interviews were scheduled to accommodate the availability of participants. The Research Ethics Boards at St. Michael's Hospital and The University of Toronto approved the study.

### Interviews

We conducted three focus group interviews (with three to five participants) and five individual interviews. Interviews were conducted by the first author and were structured to understand participants' experiences with PrEP over time, covering: (1) *initial decisions to start PrEP*, (2) *experiences of getting PrEP access both before and after the demonstration project*, (3) *sex lives and sexual decision-making on PrEP*, (4) *challenges in relation to PrEP use*, (5) *decisions about continuing to use or not use PrEP*; and (6) *participants' policy recommendations related to PrEP use and access*. Interviews were digitally audiotaped, transcribed verbatim, and reviewed for accuracy. Individual interviews lasted between 45 and 70 min, and focus group interviews lasted between 75 and 110 min. The same interview guide was used for both individual and focus group interviews. Study participants received an honorarium of $25 CAD. Participants also completed short quantitative sociodemographic and behavioral surveys immediately before commencing the interview.

### Data analysis

QSR NVivo 11 qualitative software was used to manage and organize the anonymized transcripts. We used a Grounded Theoretical approach to code and interpret the qualitative data.^20,21^ Five interview transcripts (three focus group interviews and two individual interviews) were initially reviewed and open coded by members of the research team with qualitative research experience. For focus group transcripts, this included coding for both individual participant responses and writing analytic memos to account for the dynamic exchanges between participants. Our coding strategy allowed us to inductively learn from men's narrative accounts as we systematically reviewed and open coded interview transcripts to examine the diverse experiences of our study participants. These codes were reviewed, modified, and a project code book was subsequently standardized and applied to all transcripts. Both during and after coding, short memorandums were written to capture emergent findings and inform subsequent phases of data collection and analysis.^22^ Variability in coding and interpretation was discussed through team meetings to resolve discrepancies.

## Results

We interviewed 16 gay, male-identified participants who were part of the PREPARATORY-5 demonstration project in Toronto. See Table 1 for the self-described sociodemographic characteristics of the participants interviewed for this analysis. Overall, the profile of these 16 participants was broadly similar to that of the parent PREPARATORY-5 trial, which enrolled mostly gay-identified (94%) white (73%) men, with median age 33 years, of whom 73% had at least one postsecondary degree.

**Table 1.** Self-Identified Sociodemographic Characteristics of Participants

	*PrEP trial participants (*n* = 16)*	
	N	*%*
Age group (years)		
20–29	4	25.0
30–39	7	43.8
40–49	2	12.5
50–60	3	18.8
Ethnicity		
Asian	1	6.3
Black	1	6.3
Indigenous	1	6.3
White	11	68.8
Other	2	12.5
Assigned sex at birth		
Male	16	100.0
Gender identity		
Male	16	100.0
Sexual orientation		
Gay	16	100.0
Education		
High school	1	6.3
College or undergraduate degree	10	62.5
Graduate or professional degree	5	31.3
Annual income		
<$20,000	2	12.5
$20,000–$39,999	4	25.0
$40,000–$59,999	3	18.8
$60,000–$79,999	2	12.5
>$80,000	5	31.3

PrEP, pre-exposure prophylaxis.

Rather than stigma or shame, many participants discussed being “proud” and “liberated” because of their PrEP use: “I feel proud because I know that I am doing something positive. […]. I know that I am doing the right thing by keeping myself and other people healthy” (Individual Interview). This idea of pride in being a *responsible* sexually active gay man and helping to prevent the spread of HIV was a recurrent theme in men's accounts.

In another example of the overall positive impacts of PrEP on gay men's lives, a second participant explained:

Frankly, it's been one of the greatest things of my life. I have absolutely loved it. I have a lot of sex, and I go to the bathhouses a lot, despite my advanced age. I can tell you, sex has never been better. For the first time in my lifetime, it's taken away the fear from having sex. Sex isn't meant to be something you're ashamed of or fearful of. It's meant to be enjoyable and PrEP has made sex enjoyable for me, which is fantastic. […] Now that I can have bareback sex again, it's just fantastic. Sex has been liberating again thanks to PrEP. (Focus Group)

This rich excerpt illustrates the self-described significance of PrEP on one man's life, and how—perhaps not surprisingly—one important result of PrEP use was the ability to have “liberating” condomless sex. This participant described being liberated from a fear of contracting HIV and from needing to use condoms. Many participants talked about PrEP liberation for gay men as analogous to the availability of the birth control pill for women. In these comparisons, only a small minority discussed the sex-negative attitudes associated with oral contraceptives historically.

While the experiences of PrEP use were described as liberating and empowering overall, sex-stigma emerged as a complex theme in the accounts of PrEP users. In the Results sections that follow, we describe the theme of PrEP-related stigma in men's accounts across three overlapping domains: (1) *PrEP-related stigma*, including discussions of concealment, resilience, and stigma in relation to friends, family, and sexual partners, (2) *PrEP as a perceived tool for combating HIV-related stigma*, where in some cases men said that they no longer discussed HIV status with sexual partners, and (3) *PrEP as an illuminator of structural stigma*, where PrEP was discussed as revealing or unmasking stigma related to sex and sexuality.

### PrEP-related stigma and the PrEP closet

In their accounts of stigma, a key overarching assumption men described was that for many people in their social and sexual networks, PrEP use was equated with having bareback or condomless anal sex. Men described how they have had to manage this assumption/expectation regardless of whether or not they are (exclusively) having condomless sex while on PrEP. For example, here we see how participants described what they said people assumed about their sex lives—or what they imagined that others would assume—knowing that they were on PrEP:

R1: …there were several instances in which I had to calm people down once I told them that I was on PrEP because they assumed that my whole lifestyle is changed. […] one of the regulars that I had previous to going on PrEP decided to stop doing me because he assumed that I would instantly become like a receptacle for…

R2: Yeah… [crosstalk]

R1: …for every gay plague known to man. So I mean that was kind of an uncomfortable conversation, but I was not ashamed about it. I was annoyed […] and then I was mentally preparing myself for having to have that conversation a lot of times but it turned out that he was the first and last. (Focus Group)

Some participants described how PrEP-related stigma, or stigma related to having multiple sexual partners, has led them not to tell friends and/or family about PrEP use, creating a kind of *PrEP closet*:

I hardly told anybody, just because I know my friends would be judgmental—gay or straight. For someone who is as out as me, and I'm about as out as anybody, to not admit I'm on PrEP, it's weird. It's like there's still a stigma attached to it, which is totally unfair. (Focus Group)

For another participant, who had recently opened up his relationship to other sexual partners beyond his husband, talking about PrEP use was seen as exposing his nonmonogamous sexual practices (as well as those of his husband), which were thought to be stigmatized sexual activities especially in the context of marriage:

I don't disclose that I am on PrEP to most family and most friends. That's maybe because I am married and I have kids, so for his [husband's] sake, I am not really, we are not open—fully open that we're open if you know what I mean. So I mean I should be able to be, but I don't think we're there yet. So I wouldn't say that I feel ashamed for it but I definitely have to hide it… (Focus Group)

Some men discussed experiences of PrEP-related judgment, stigma, or rejection when trying to connect online with prospective sexual partners. For example, the participant above described only one negative experience, noting that he was able to easily “brush it off”: “Just the one person on Grindr, but that was a complete stranger so I just brushed it off” (Focus Group). Using similar language, another participant recounted experiencing stigma and rejection from friends, which prepared him for subsequent stigmatizing experiences from prospective sexual partners:

I just sort of blew it off or blow it off. It's like I think after my two very early arguments of my best friend and one with a friend who broke the friendship; after that I think I was prepared for any kind of cursedness like ‘Oh, you're on PrEP, I'm not going to sleep with you, because you're clearly a slut’ and I'm like ‘you don't even understand what it's about.’ That's fine, whatever. (Individual Interview)

### PrEP and HIV-related stigma

Extending the discussion in the last section, some men talked about both condomless sex between men being stigmatized and sex with gay men living with HIV. In their accounts, participants discussed how their PrEP use caused them to reflect on and change or challenge internalized stigma or taboos they had in relation to persons living with HIV. For example, one man explained: “…I grew up in a time where the notion of unprotected sex was not only stigmatized, but people wouldn't engage sexually with people that were HIV positive even with condoms, right?” (Individual Interview). This participant continued to discuss his PrEP use as *empowering* in the context of his relationship with a partner who was living with HIV, including why his partner's undetectable viral load was not enough to make him feel protected: “No, it's because I am in control of my health versus what he is doing for his health which is again a silly argument but that's how I feel better.”

Despite reporting knowledge that undetectability means having virtually no risk of HIV transmission, participants described relying solely on their partner's viral load as a contingency based on the actions and adherence of another person (even if this was a primary sexual partner). Men elucidated how PrEP use allowed them to be *in control* and do something *for themselves* to reduce their risk of HIV.

Some participants discussed stigma toward HIV-positive men and how PrEP opened them up to having “better” or “more honest” sex with guys overall, including those who had been diagnosed with HIV. Here we see a participant discuss his sex with men living with HIV:

I mean I know there is a lot of stigma toward positive guys but when I had sex, I felt like most of the time it was better. You know that's, you know, and I built like good relationships, like I have—I made many friends who are undetectable or positive. […] I just felt like you know we were both honest and he was honest and you know. […] Whereas like if I had sex with a negative guy, it is—actually guys who aren't on PrEP, it would, I don't know it's just different. It's hard to explain. It's not like…I find them both good, I find like sex is good for both. (Individual Interview)

In a related narrative, another participant explained what he saw as the persistent “moral stigma” related to HIV and the significant way he saw PrEP as combating stigma in this regard.

Some men also discussed their perceptions that PrEP lessened feelings of stigma and rejection among gay men who are HIV positive:

…it kind of made me feel good I don't have to ask guys what their status is and so poz guys don't have to worry about disclosure or rejection and you know all that stigma stuff, and I like the idea that they have a different experience now that there are so many guys on PrEP because some guys would just—like I don't ask. (Focus Group)

For this participant, the potential of PrEP to destigmatize HIV took the form of him choosing to no longer discuss HIV status with partners.

### PrEP and structural stigma

Finally, participants described how PrEP sometimes exposed broader structural forms of stigma related to sex and gay sexuality. In some cases, stigma related to PrEP and gay sexuality was discussed as an insidious structural barrier to broader PrEP awareness and access. For example, in one focus group, participants said the following when asked about their policy recommendations related to PrEP use and access:

R1: I would start with education. I think there's a huge segment of the gay community that doesn't know about PrEP. That would be my number one thing. Number two would be cost. Until it comes down in price, or the equivalent drug comes down in price, it'll be prohibitive and you'll never get people on it.

R3: […] We now have this tool that we can use to virtually eliminate new infections in our major cities and everywhere, and those are savings for the health care system by reducing and removing HIV that would be incredible.

R1: If somebody invented an anti-cancer drug, people would say that it is a miracle, regardless of it costing $1000 a month. Because it's an HIV treatment, there's still a stigma. Gay men just get it [HIV] because they're fooling around. That's not true at all. I think that hurts too.

R3: I think there's a real case to be made for the equality argument, equality of access to healthy sex that straight people already have. (Focus Group)

Again, here we have a rich passage where focus group participants spoke about their belief that stigma related to sex and gay sexuality in particular is a pervasive barrier to PrEP access.

Some participants described encountering judgment both from within their social and sexual networks and from some healthcare providers during their initial attempts to access PrEP. For example, a number of men expressed barriers to accessing PrEP from physicians due to feelings of discomfort related to discussing PrEP, gay sexuality, and sexual risk with doctors. In some of the exchanges between focus group participants, talk of pride in being an early adopter of PrEP contrasted with accounts of having to resist pervasive stigma: “There's definitely a stigma. You can't get around it if you take Truvada. People just judge you. I don't have any personal shame but people do judge” (Focus Group).

Some men described that they felt they had a responsibility to educate others about PrEP, noting that broader education in and beyond the gay community in Canada was needed to help overcome PrEP access and literacy barriers. The case for equality—*and equal access to healthy, pleasurable sex*—was made my numerous participants across both focus groups and individual interviews.

## Discussion

The participants in our study were early adopters of PrEP in Canada and, as such, on the frontlines of both PrEP use and some forms of PrEP-related stigma. However, rather than talking about being overly burdened by stigma or shame, many participants discussed being “proud” and “liberated” because of their PrEP use. This was not a “spoiled identity” in need of stigma management,^23^ but rather an innovation to celebrate and, for some, a *return to normalcy*—normal, exciting, pleasurable sex that many deemed as no longer reliant on condom use. Some men drew connections to the current availability and normalization of the oral contraceptive pill for women.^24^ Our point here is not to critique this practice of condomless sex on PrEP, nor to assess men's practices of “risk compensation,”^25^ but instead to recognize that for many participants, sex free of condoms was one significant reason PrEP was said to have positively impacted their lives. PrEP has allowed some gay men to be more open about the kinds of sex they want to have and enjoy condomless anal sex.^*^

The narratives we have drawn upon make explicit how PrEP was described in relation to stigma in complex ways: PrEP being both a kind of (internalized) *stigma fighter* and something linked to experiences of stigma and sexual morality, recapitulating some gay men's experiences with having “something to hide” regarding their sexuality. Accounts of PrEP concealment revealed a kind of *PrEP closet* for some participants.^26^ Men's accounts of PrEP use and disclosures or concealments in relation to this biomedical intervention shed light on underlying pernicious challenges, tensions, and stressors related to gay sexuality.^27^ Our findings link to the commentary of Race who discusses the slow uptake of PrEP and argues that “Despite its proven efficacy as an HIV prevention strategy among men who have sex with men, PrEP has so far emerged as a *reluctant object*, partly because of its putative association with the supposed excesses of unbridled sex.”^28^

Most men were reluctant to assume a victim narrative, or frame PrEP in a negative light, noting that not only were they able to “manage” the negative issues associated with PrEP but that stigma *rolled off their back* so-to-speak. Our participants described how PrEP removed fear and stress related to contracting HIV, and told us that they saw PrEP as representing significant social progress for HIV-positive guys that helped to bridge the “sero-divide.”^29,30^ However, we think it is important to reflect on the public health significance of some men on PrEP saying that they no longer discuss HIV status with sexual partners because of their PrEP use. While this reported change in behavior may be intended as a form of social progress—aimed at destigmatizing HIV positivity—there may also be potential unintended consequences of this behavior if more widespread. We feel that clinical counselors and PrEP educators should be mindful of this potential change in behavior as PrEP rollout expands.

We have elucidated a paradox in participant's accounts: men said that PrEP use led them to *experience stigmatizing reactions* within their social and sexual networks while also being described as helping to *remove stigma*, *shame*, *and fear* related to HIV, sexuality, and sex with gay men living with HIV. We believe it is essential that these narratives be read with reference to gay men's political struggle and historical trauma, with an understanding of the evolving policies and discourses that coordinate and regulate gay men's sexual and social lives. Successfully advocating for broader PrEP access requires that societal and structural stigma surrounding gay sexuality be addressed head on. These accounts of PrEP use help to shed light on broader stigmas and moral panics around sex and sexuality, which may serve to negatively impact both the availability and uptake of PrEP and the health of diverse communities, including, although certainly not limited to, gay men.

Our study is subject to several limitations. It is important to note the relative social and economic privilege of many of the participants interviewed. For example, this was a small sample of largely white, highly educated gay men. In being highly motivated “early adopters,” our sample may have been more likely to experience and/or report an optimistic or positive outlook on PrEP. It is possible that future users may not be as inclined to see PrEP as something positive for gay men living with HIV, or may not be as likely to have stigmatizing exchanges related to PrEP and gay sexuality *roll off their back*.

More research with gbMSM across diverse *intersectional* geographies and social locations,^31,32^ including people of color,^33,34^ non-cisgender, and non-gay identified gbMSM, may reveal differing experiences of PrEP access, management, and experiences of stigma related to PrEP and sexuality. We also believe further research is necessary to understand the experiences of other communities who are using or may benefit from PrEP, including those in resource-poor countries,^35^ as well as gbMSM who have worked to gain access to PrEP outside of the context of trials/demonstration projects. Considering the experiences and perspectives of gbMSM living with HIV^36,37^ in the context of an evolving biomedical prevention landscape is also important to understand given the stigma-reducing attributions to PrEP that were articulated by our sample of HIV-negative participants.

## Notes

^*^ Based on in-depth qualitative interviews with a sample of 40 PrEP users in Toronto and Ottawa, Canada, we are currently completing an analysis of men's accounts of sexual behaviors on PrEP, including how PrEP has altered previous seroadaptive behaviors.^9^
